# UV Radiation Induces Specific Changes in the Carotenoid Profile of *Arabidopsis thaliana*

**DOI:** 10.3390/biom12121879

**Published:** 2022-12-14

**Authors:** Uthman O. Badmus, Gaia Crestani, Natalie Cunningham, Michel Havaux, Otmar Urban, Marcel A. K. Jansen

**Affiliations:** 1 School of Biological, Earth and Environmental Sciences & Environmental Research Institute, University College Cork, Distillery Fields, North Mall, T23 N73K Cork, Ireland; 2 Bioscience and Biotechnology Institute of Aix-Marseille, Aix-Marseille University, CEA, CNRS, UMR7265, CEA/Cadarache, F-13115 Saint-Paul-lez-Durance, France; 3 Laboratory of Ecological Plant Physiology, Global Change Research Institute, Czech Academy of Sciences, Brno, Belidla 4a, CZ-60300 Brno, Czech Republic

**Keywords:** carotenoid, xanthophyll, photoreceptor, photosynthesis, arabidopsis, UV-B

## Abstract

UV-B and UV-A radiation are natural components of solar radiation that can cause plant stress, as well as induce a range of acclimatory responses mediated by photoreceptors. UV-mediated accumulation of flavonoids and glucosinolates is well documented, but much less is known about UV effects on carotenoid content. Carotenoids are involved in a range of plant physiological processes, including photoprotection of the photosynthetic machinery. UV-induced changes in carotenoid profile were quantified in plants (*Arabidopsis thaliana*) exposed for up to ten days to supplemental UV radiation under growth chamber conditions. UV induces specific changes in carotenoid profile, including increases in antheraxanthin, neoxanthin, violaxanthin and lutein contents in leaves. The extent of induction was dependent on exposure duration. No individual UV-B (UVR8) or UV-A (Cryptochrome or Phototropin) photoreceptor was found to mediate this induction. Remarkably, UV-induced accumulation of violaxanthin could not be linked to protection of the photosynthetic machinery from UV damage, questioning the functional relevance of this UV response. Here, it is argued that plants exploit UV radiation as a proxy for other stressors. Thus, it is speculated that the function of UV-induced alterations in carotenoid profile is not UV protection, but rather protection against other environmental stressors such as high intensity visible light that will normally accompany UV radiation.

## 1. Introduction

In the natural environment plants are subjected to a complex spectrum of light that includes, amongst others, ultraviolet (UV; 280–400 nm), visible (400–700 nm), and far-red (FR; 700–780 nm) wavelengths. The quality and quantity of light are crucial in controlling plant growth and development [1,2]. UV-B (280–315 nm) is an integral part of the solar spectrum, although in the natural environment wavelengths shorter than 290 nm are not normally detectable. UV-B is considered a regulator and/or stressor depending on the intensity [3]. As a regulator, UV-B triggers multiple protective responses in plants such as the induction of antioxidant defences and photorepair capacity, accumulation of a broad range of metabolites and changes to plant morphology [4,5,6,7]. Conversely, as a stressor, UV-B exposure can reduce photosynthetic efficiency, and cause Reactive Oxygen Species (ROS) production and DNA damage [8,9,10]. UV-A radiation (315–340 nm) is less reactive on a per photon basis but can also cause both inhibitory and stimulatory effects on plants [11].

There is a wealth of knowledge on the relationship between UV-exposure and metabolite accumulation in plants. Reports have been published on the UV-induced accumulation of metabolites such as flavonoids, glucosinolates, anthocyanins, tocopherols, and polyamines, on changes in expression of key metabolic genes, on the functional role of UV-induced metabolites and on the roles of photoreceptors in metabolic reprofiling [12,13,14,15,16,17,18,19,20]. Based on the complexity of the plant metabolite network, including cross-talk between different metabolic pathways [21] and/or acclimation pathways [22], it can be hypothesised that effects of UV will extend across a broad range of metabolite classes. However, at present the UV responses of some classes of metabolites are not well studied. One example of a class of metabolites of which the response to UV-exposure is not fully understood, is the carotenoids [23].

**Figure 1:**
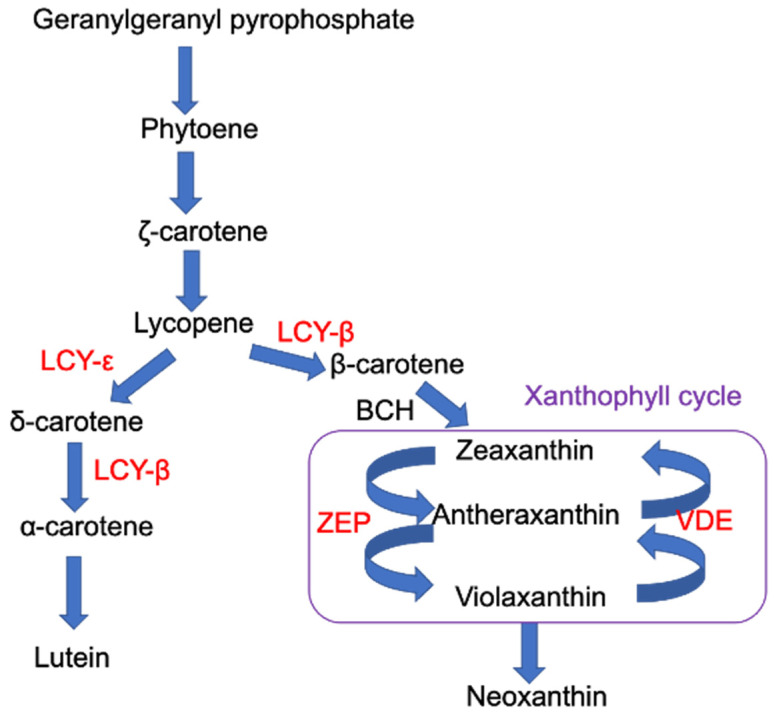
Simplified plant carotenoid biosynthetic pathway. Text in red refers to the enzymes LCY-ε (lycopene epsilon cyclase), LCY-β (lycopene beta cyclase), BCH (carotenoid beta-ring hydroxylase), ZEP (zeaxanthin epoxidase), VDE (violaxanthin de-epoxidase)) that catalyse various steps of the carotenoid pathway. Mutants in several of these genes were used in this study as specified in Table 1 (ZEP, aba1; LCY-ε, *lut1*; VDE, *npq1-2*; LCY-β and VDE, *szl1-1npq1-2*).

Carotenoids are a diverse class of metabolites belonging to the family of terpenoids. These lipid-soluble compounds include xanthophylls that are oxygenated, and carotenes that are not. In higher plants, carotenoids are mainly found in the chloroplast and are synthesised by combining isopentenyl pyrophosphate with dimethylallyl pyrophosphate to form geranylgeranyl pyrophosphate (GGPP). The latter reacts with another GGPP in the presence of phytoene synthase to form phytoene [24,25]. Phytoene is then desaturated (in the presence of phytoene and ζ-carotene desaturases), and isomerised (using carotene isomerase) to lycopene (Figure 1). Lycopene is a central intermediate in the biosynthesis of carotenoids and can be converted into either β- or δ-carotenes, in the presence of lycopene-β-cyclase or lycopene-ε-cyclase, respectively. These two carotenes are the precursors for the formation of all other carotenoids [24,25], i.e., δ-carotene can be converted in to α-carotene and lutein, while β-carotene can be converted in to zeaxanthin, antheraxanthin, violaxanthin and neoxanthin [24,25,26,27].

Carotenoids are important plant metabolites controlling numerous plant physiological functions. These pigments are accessory photosynthetic pigments, and play a key role in the photoprotection of the photosynthetic machinery by quenching triplet excited chlorophylls and ROS. Carotenoids are also important structural components in the formation and dynamics of antenna and reaction centre [24,25,26,27,28,29,30,31,32,33]. Within the carotenoids, both distinct as well as overlapping functions can be identified. For example, lutein and β-carotene can both quench singlet oxygen; lutein in the antennae, and β-carotene in the photosystem reaction centres [34,35]. Lutein can also quench the triplet chlorophyll state thus avoiding formation of singlet oxygen, while it is thought that β-carotene cannot quench triplet chlorophyll for topological reasons (located distant from chlorophyll) [28,36,37]. Perhaps the best-known role of carotenoids in photosynthesis relates to the dissipation of excess light energy through the xanthophyll cycle. This involves the xanthophylls zeaxanthin, antheraxanthin and violaxanthin which mediate a process called non-photochemical quenching [36,38,39].

The role of carotenoids in the plant is not limited to photosynthesis, as carotenoids contribute to the regulation of plant growth and development through the carotenoid-derived phytohormones abscisic acid (ABA) and strigolactone [40,41,42] and the apocarotenoid regulators β-cyclocitral or zaxinone [43]. Additionally, carotenoids play a key role in plant ecological interactions, for example through colouration of flowers, fruits as well as roots [24,44]. Furthermore, carotenoids can move up along the food chain to higher trophic levels, where they can play important physiological and ecological roles [45], amongst others by determining the development of colours in, amongst other, bird plumage and fish skin [46]. Thus, the quantitative accumulation of carotenoids has wide ranging consequences for plants and wider ecosystems.

There is ample evidence concerning the visible light induced synthesis and accumulation of carotenoids in plants as well as their role in photoprotection [27,28,33,38,47]. In comparison, the induction of carotenoids by UV radiation, and a potential role in UV protection are not well established. It was reported [48] that high UV-B doses upregulate carotenoid biosynthesis only in the short term, suggesting that carotenoids might form part of a transient (days) UV response. A recent meta-analysis by Badmus et al. [23] captured data from 18 published studies, both short-term and long-term exposures, and concluded that across the entire data-set violaxanthin was the only carotenoid compound significantly induced by UV radiation. Remarkably, this was accompanied by a lowering of antheraxanthin and zeaxanthin contents, resembling adjustment of the xanthophyll cycle to low light conditions. Perhaps this phenomenon is associated with the reported UV-B mediated inhibition of the activity of violaxanthin de-epoxidase activity in plants [49]. Yet, in direct contradiction with the results of the recent meta-analysis, a study on Arabidopsis reported UV-B mediated induction of zeaxanthin accumulation [50]. At present, the reason for such contradictory data is not understood.

Overall, UV-mediated carotenoid accumulation is not well characterised. Questions remain concerning the regulatory mechanism, and especially the relevance of photoreceptors. Questions also remain concerning the functional role of UV-induced carotenoids in UV protection. A study by Emiliani et al. [50] reported that a general reduction of carotenoid content (as well as tocopherols) resulted in increased UV-B sensitivity, measured as DNA damage and impairment of photosynthesis. In this study, the induction of carotenoids was determined in wild-type (Col-0) *Arabidopsis thaliana* exposed to UV for up to 10 days. In parallel, accumulation of carotenoids *was* determined in *Arabidopsis thaliana* mutants lacking the functional photoreceptors (*uvr8*, *cry1cry2* and *phot1phot2*) or impaired in carotenoid biosynthesis. It was hypothesised that only specific carotenoids would accumulate in UV-exposed plants, and that the functional role of these upregulated carotenoids could be ascertained through the study of Arabidopsis mutants.

## 2. Methodology

### 2.1. Plant Material

*Arabidopsis thaliana* wild-type (Col-0) and Col-0 background photoreceptor mutants were studied. These include the UV-B photoreceptor mutant *uvr8* [51], the cryptochrome double mutant *cry1cry2* [52], and the phototropin double mutant *phot1phot2* [53]. Seeds of *phot1phot2*, *aba1-6*, *lut1*, *npq1-2*, *npq4-1* and *szl1-1npq1-2* were acquired from NASC, Nottingham, United Kingdom. Professor Roman Ulm (University of Geneva, Geneva, Switzerland) kindly donated *uvr8* and Professor Stephan Wenkel (University of Copenhagen, Copenhagen, Denmark) the *cry1cry2* line. Furthermore, the carotenoid Col-0 background mutants *aba1-6*, *lut1*, *npq1-2*, *npq4-1* and *szl1-1npq1-2* were used for this study (Table 1). Seeds were plated in a Petri dish containing a wet filter paper (Whatman, Kent, UK), which was sealed with parafilm and placed in a fridge at 4 °C for 72 h. Seeds were then transferred to Jiffy plugs (Deker Horticulture, Co. Meath, Ireland) that were pre-soaked in distilled water. Plugs were transferred into transparent plastic trays which were covered with cling film and placed under cool white fluorescent light photosynthetic active radiation (PAR) (36W Philips Master TLD Reflex Tube, BLT Direct) at 70 μmol m^−2^ s^−1^ with a 16/8 h light/dark regime at 21/18 °C and 60% relative humidity in a growth chamber. Plugs were watered at least twice a week. No additional fertiliser was used.

### 2.2. UV Treatments

On attaining the 1.08 plant developmental stage [58] after 16 days, plants were subjected to UV treatment (TL-12 40W, UV-B broadband, Philips). UV tubes were wrapped with cellulose acetate films (95 µm thickness; Kunststoff-Folien-Vertrieb GmbH, Hamburg, Germany) to exclude UV-C, and mounted 30 cm above the plants. UV tubes were on for 3.75 h daily between 12:00–15:45. UV controls were generated by placing boxes wrapped with UV-B blocking mylar film (125 µm thickness, Tocana Ltd., Dublin, Ireland) over plants while boxes with cellulose acetate UV-B transmitting acetate film were placed over UV treated plants. Plants covered with mylar are exposed to both PAR and a small amount of long wavelength UV-A radiation, and these are referred to as untreated controls. Plants covered by cellulose acetate are exposed to PAR, long and short wavelength UV-A and UV-B, and are referred to as UV-treated. Under the cellulose acetate filter, the UV (i.e., 280–400 nm) irradiance was 0.59 W m^−2^ of which 0.22 W m^−2^ UV-B (280–315 nm) (Table 2). The calculated biological effective daily dose using the action spectra formulae Gen-T was 2.79 kJ m^−2^, of which the UV-B wavelength band comprised 2.53 kJ m^−2^ [59]. The used UV dose was kept relatively low compared to ambient solar UV doses, as low intensities of background PAR can artificially enhance UV sensitivity. UV quality and intensity were regularly determined with a FLAME-T-UV-VIS-ES Spectrometer (Ocean Insights, Duiven, The Netherlands) and PAR meter (SKR100, Skye instruments, Wales, UK). UV was accompanied by 188 μmol m^−2^ s^−1^ PAR with a 14/10 h light/dark regime, generated by LED lamps (AP67 R-series, Valoya, Finland) supplemented with far-red tubes (RoHS, L18 spectrum FR730, Valoya, Finland) to take the R: FR to 1.6. The background intensity of PAR was selected as it resulted in good plant growth, with no evidence of macroscopic UV damage to plants.

For the carotenoid induction (kinetic) study, plants were exposed to UV for up to 10 days. Initially, plants were harvested immediately after UV-exposure, and again 18 h later. As no differences were noted between the two sets of plants, the protocol was standardized at a harvest 18 h after UV-exposure. Thus, plants were harvested on the 2nd day at 10 am (i.e., before the UV application on that day), and similar pre-UV-exposure sampling was undertaken on days 4, 6, 8 and 10. The induction study revealed that a strong alteration of carotenoid profile could be obtained following 3 days of daily UV-exposure plus 18 h of recovery, i.e., on the 4th day at 10 am. Therefore, this timepoint was selected for the photoreceptor and carotenoid biosynthesis mutant studies. Harvested plant material was immediately pulverised using liquid nitrogen and transferred into 2 mL Eppendorf tubes before storing at −80 °C. A total of 5 independent biological replicates were achieved by growing plants and applying UV in the same room on separate occasions.

### 2.3. Chlorophyll a Fluorescence Analysis

Chlorophyll *a* fluorometry was undertaken using a pulse amplitude modulated WALZ Imaging fluorometer (Walz, Effeltrich, Germany). Chlorophyll *a* fluorometry was used to measure the maximum quantum yield of photosystem II (PSII) (F_v_/F_m_) in whole plants dark-adapted for 20 min. Subsequently, plants (18 h after UV exposure) were exposed to an actinic light intensity of 186 μmol m^−2^ s^−1^, i.e., a PAR intensity similar to that during UV-exposure experiments, till photosynthesis reached a steady-state. At this stage, the effective quantum yield of PSII (Y(II)), the proportion of energy being dissipated through regulated non-photochemical quenching (Y(NPQ)), and the proportion of energy being dissipated in a non-regulated non-photochemical manner (Y(NO)) [60] were measured. Y(NO) is associated with non-regulated heat dissipation and fluorescence emission, and together with Y(NPQ) and Y(II) equates one.

### 2.4. Carotenoid and Chlorophyll Extraction

Carotenoids were extracted using a modification of the method described in [61]. Briefly, 50 mg of Arabidopsis leaves (entire rosette) that had been ground in liquid nitrogen was weighed into 2 mL Eppendorf tubes, and 1000 μL of cold acetone was added. The tube was homogenised with a plastic pestle, vortexed, and sonicated in cold water for 20 min. After which, the tube was vortexed and centrifuged at 13,000 rpm for 10 min at room temperature. The acetone organic phase was collected into a separate tube and the plant material was reextracted by adding 500 μL acetone and vortexing and centrifuging were repeated. Extracts were pooled together, filtered using a 0.45 µm pore size PTFE syringe filter (Fisher Scientific, Dublin, Ireland), transferred into an amber glass vial and analysed on HPLC.

### 2.5. Carotenoid and Chlorophyll Separation Using HPLC

Carotenoids were quantified using a high-performance liquid chromatography (HPLC) 1290 infinity II system with both 1260 Infinity II Diode Array Detector and Fluorescence detectors, a multicolumn and autosampler (Agilent Technologies, Santa Clara, CA, USA). The analytical method was adapted from [50,62]. The analytical column used was a carotenoid C-30 reverse phase column (YMC America, Devens, MA, USA) (250 mm length, 4.6 mm inner diameter, 3 μm resin diameter), with a flow rate of 1 mL min^−1^ and injection volume of 10 μL. The mobile phase consisted of methanol (solvent A), 80% MeOH with 0.2% ammonium acetate (*v*/*v*) (solvent B) and tert-methyl butyl ether (solvent C). The column was eluted using the following gradient: solvent A:B:C ratio at 0 min was 95:5:0 and was held for 12 min (12 min) before moving to 80:5:15 until 24 min and to 30:5:65 until 30 min. Afterwards, elution returned to 95:5:0 for 20 min, giving a total elution of 50 min. The column was kept at 18 °C and the diode array detector was set to collect data between 190–700 nm, UV peaks were monitored at 280 and 450 nm for carotenoids and 650 nm for chlorophylls. Identification and quantification of individual chlorophylls and carotenoids were achieved using commercially acquired pure standards. A lutein standard was used for quantifying zeaxanthin, antheraxanthin and β-carotene due to a lack of pure analytical standards. Commercial β-carotene standards tend to contain some α-carotene and were not used. Lutein, violaxanthin and neoxanthin, and chlorophyll *a* and *b* standards were acquired from Sigma Aldrich. The carotenoids quantified were the most common compounds in the samples, and include antheraxanthin (Ant), neoxanthin (Neo), violaxanthin (Vio), lutein (Lut), zeaxanthin (Zea), β-carotene (β-Car), VAZ (sum of Vio, Ant and Zea), Total carotenoid (sum of all carotenoids) and de-epoxidation state of xanthophyll cycle (AZ/VAZ; DEPS) [8].

### 2.6. Statistical Analysis

A two-way ANOVA was utilised to determine the effect of treatment (UV versus control) versus treatment days (i.e., duration) and/or genotypes, using AOV in the package *stats*. As normality of variances could be assumed for all variables, all data (+UV versus −UV) were compared using pairwise *t*-tests at each treatment day and genotypes, respectively, using the package *rstatistix*. Significance was adjusted for multiple testing with the BH correction and bar plots were plotted using the package *ggpubr*. Principal Component Analysis (PCA), to visualise the relationship between carotenoids and photosynthetic parameters, was performed using the *prcomp* function in package *stats* [63]. All statistical analyses were performed on R (version 4.0.53 in RStudio (version 1.4.1103)). Data and codes used for the analysis are shown at https://github.com/LanreBadmus/UV_Carotenoid_Induction., (accessed on 13 December 2020)

## 3. Results

The UV-mediated induction of carotenoids in *Arabidopsis thaliana* (Col-0) plants was studied as a function of UV-exposure duration, followed by an analysis of inductive responses in photoreceptor and carotenoid biosynthesis mutants.

### 3.1. UV-Induced Carotenoids in Arabidopsis Thaliana

Quantification of carotenoid contents in untreated (no UV-exposure) Arabidopsis shows that Lut is the most abundant carotenoid at an average content of 105 µg·g^−1^ FW, followed by Vio with an average of 54 µg·g^−1^ FW.

**Figure 2:**
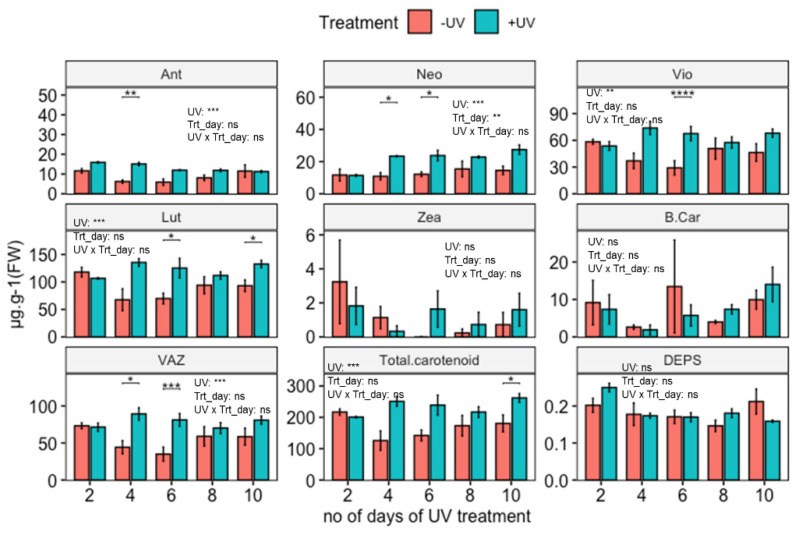
Carotenoid contents (µg·g^−1^ FW, except DEPS) of wild-type *Arabidopsis thaliana* (Col-0) exposed to varying durations of UV. Plants were subjected to UV treatment for 3.75 h daily and harvested after 18 h on days 2, 4, 6, 8 and 10. Bar plots show means and error bars indicate standard error for n = 5. The text in each panel shows the outcome of a two-way ANOVA across treatment and exposure duration. The stars above pairs of bars show the outcome of *t*-tests between the UV treatments. In both cases * (*p* < 0.05), ** (*p* < 0.01), *** (*p* < 0.001), **** (*p* < 0.0001), depict significant differences with ns depicting a non-significant difference.

UV-induced carotenoid accumulation was observed and for Neo found to be time-dependent (Figure 2). The induction process is time-dependent, and the quantitative degree of induction changed as the duration of the UV application increased. A two-way ANOVA shows significant increases in Ant, Vio, Neo, Lut, VAZ and total carotenoids. Subsequent paired *t*-tests show significant induction of Lut, Vio, Neo and VAZ for plants exposed to UV for 4, 6 and 10 days. In the case of Ant, relative induction was highest for plants exposed to 2, 4, or 6 days of UV. The sum of all carotenoids (Total carotenoid) with an average content of 201 µg·g^−1^ FW, showed UV induction for all exposure durations, with a significant effect on plants exposed to UV for 10 days. Despite apparent peaks in the contents of Zea and β-Car on day 6, statistical analysis did not reveal significant UV induction (*p* > 0.05) of these two carotenoids across treatment and durations.

Some of the key differences between UV exposed and control plants are noted for days 4 and 6 (Ant, Lut, Neo, Vio, VAZ and total carotenoids). This effect comprises an increase in the content of Neo in UV exposed plants compared to when plants were first measured on day two, but a decrease in Ant in untreated controls. Contents of Vio, Lut, VAZ and total carotenoids do increase in UV exposed plants as well as decrease in untreated controls. As plants were retained in the same growth room during UV-exposure, effects on control plants are attributed to filter-effects and/or exposure to a small amount of UV-A radiation.

Alongside carotenoids, varying durations of UV-exposure also resulted in increases in chlorophyll contents in plants (Supplementary Figure S1). The most notable (and significant) increase in chlorophyll content occurred in plants exposed to UV for 6 days.

### 3.2. Influence of UV on Photosynthetic Parameters

**Figure 3:**
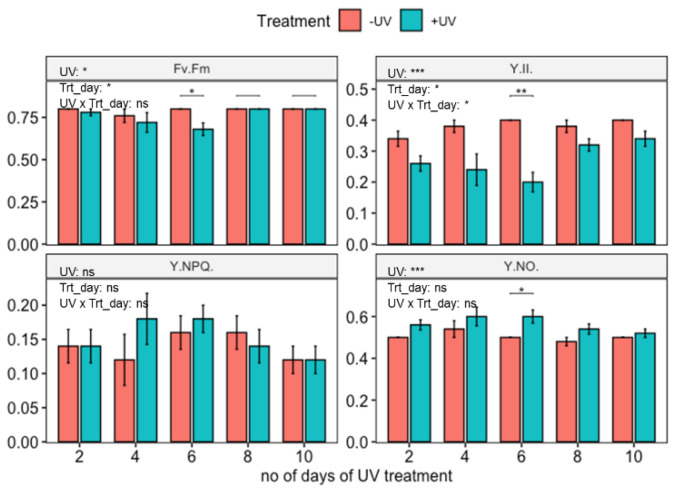
Photosynthetic efficiency of wild-type *Arabidopsis thaliana* (Col-0) exposed to varying durations of UV. Plants were subjected to UV treatment for 3.75 h daily and harvested after 18 h on days 2, 4, 6, 8 and 10. F_v_/F_m_: the maximum quantum efficiency of photosystem II (PSII); Y(II): the quantum efficiency of PSII under steady-state light conditions; Y(NO): yield of non-regulated non-photochemical energy dissipation and Y(NPQ): the yield of the regulated non-photochemical energy dissipation. Bar plots show means and error bars for n = 5. The text in each panel shows the outcome of a two-way ANOVA across treatment and exposure duration. The stars above pairs of bars show the outcome of *t*-tests between the UV treatments. In both cases * (*p* < 0.05), ** (*p* < 0.01) and *** (*p* < 0.001) depict significant differences with ns depicting a non-significant difference.

Untreated, wild-type *Arabidopsis thaliana* (Col-0) displayed a good photosynthetic efficiency (F_v_/F_m_ = 0.79 and Y(II)= 0.38) (Figure 3). In contrast, two-way ANOVA showed that plants that had been exposed to UV for up to 10 days displayed a slight reduction in F_v_/F_m_ and Y(II) as well as an increment in Y(NO), but this was significant only in plants harvested on day 6 (*t*-test). No significant impact of UV-exposure was found on Y(NPQ).

### 3.3. Role of Photoreceptors in the Induction of Carotenoids

To identify the mechanism underlying the UV-induced accumulation of carotenoids, accumulation was compared between wild-type *Arabidopsis thaliana* and genotypes lacking the functional photoreceptor *uvr8*, *phot1phot2* or *cry1cry2*. Untreated wildtype (WT) plants were kept under a mylar filter while UV treated samples were exposed to UV for 3 days before harvesting on the 4th day. Quantitative data on carotenoid accumulation showed that the carotenoid profiles of non-UV as well as UV treated photoreceptor mutants were statistically similar to those of corresponding WT Arabidopsis (Supplementary Figures S2 and S3). It is noted that untreated *cry1cry2* and *uvr8* both contain more Zea than the untreated WT, but this is not significant. Similarly, *cry1cry2* also contains somewhat more Neo, but again this is not significant.

**Figure 4:**
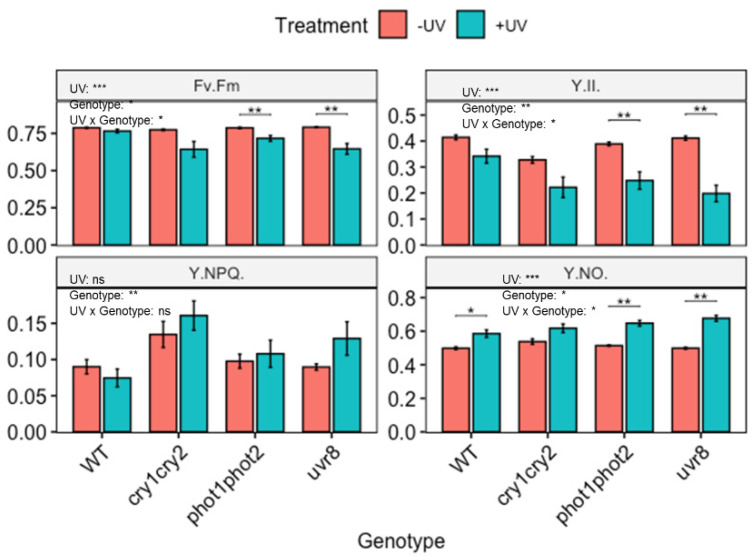
Photosynthetic efficiency of wild-type (WT) and photoreceptor deficient *Arabidopsis thaliana* (Col-0) exposed to UV; *cry1cry2* (cryptochrome), *phot1phot2* (phototropin) and *uvr8* (UV-B photoreceptor). Plants were subjected to UV treatment for 3.75 h daily and harvested after 18 h on day 4. F_v_/F_m_: the maximum quantum efficiency of photosystem II (PSII); Y(II): the quantum efficiency of PSII under steady-state light conditions; Y(NO): yield of non-regulated non-photochemical energy dissipation and Y(NPQ): the yield of regulated non-photochemical energy dissipation. Bar plots show means and error bars indicate standard error for n = 5. The text in each panel shows the outcome of a two-way ANOVA across treatment and exposure duration. The stars above pairs of bars show the outcome of *t*-tests between the UV treatments. In both cases * (*p* < 0.05), ** (*p* < 0.01) and *** (*p* < 0.001) depict significant differences with ns depicting a non-significant difference.

Despite the lack of photoreceptor associated changes in carotenoid profile in untreated as well as UV treated plants, it is noted that the photoreceptors are critical for the protection of photosynthetic machinery (Figure 4). Under control (non-UV) conditions, photosynthetic performance of photoreceptor mutants is similar to that of WT Arabidopsis, although it is noted that the *cry1cry2* mutant displays a slightly higher Y(NPQ) but lower Y(II). This effect is, however, not significant. The comparison of photosynthetic parameters between WT Arabidopsis and photoreceptor mutants following UV-exposure showed that photoreceptors play a significant role in protecting photosynthesis from UV (Figure 4). For example, in both *phot1phot2* and *uvr8*, F_v_/F_m_ was reduced in UV exposed plants. No such effect was noted in the corresponding WT. Additionally, UV-exposure significantly lowered Y(II) and increased Y(NO) in both *phot1phot2* and *uvr8* compared to untreated plants.

### 3.4. The UV-Mediated Accumulation of Carotenoids in Arabidopsis Biosynthesis Mutants

**Figure 5:**
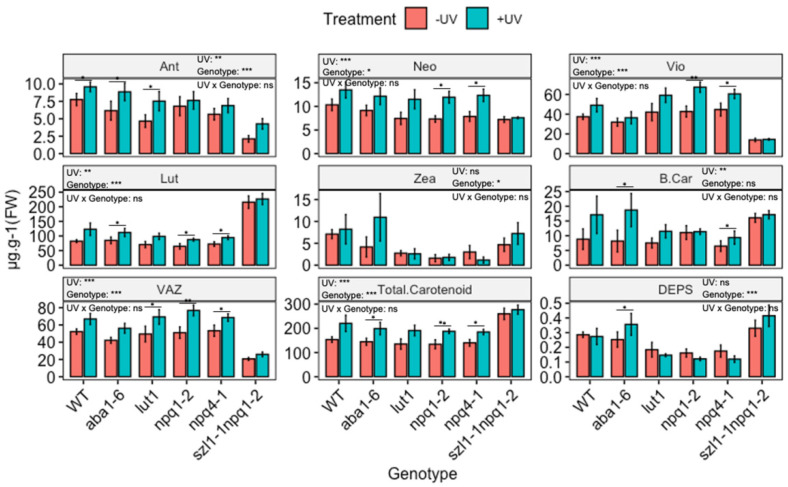
Carotenoid contents (µg·g^−1^ FW, except DEPS) of wild-type (WT) and carotenoid biosynthesis (*aba1-6*, *lut1*, *npq1-2*, *npq4-1* and *szl1-1npq1-2*) impaired *Arabidopsis thaliana* (Col-0). Plants were subjected to UV treatment for 3.75 h daily and harvested after 18 h on day 4. Bar plots show means and error bars indicate standard error for n = 5. The text in each panel shows the outcome of a two-way ANOVA across treatment and exposure duration. The stars above pairs of bars show the outcome of *t*-tests between the UV treatments. In both cases * (*p* < 0.05), ** (*p* < 0.01) and *** (*p* < 0.001) depict significant differences with ns depicting a non-significant difference.

The functional role of individual UV-induced carotenoids in protecting photosynthesis was explored by exposing plants lacking the ability to synthesise individual carotenoids to UV radiation, as shown in Figure 5. Both *npq1-2* (impaired violaxanthin de-epoxidase) and *npq4-1* (defective in pH-dependent NPQ) genotypes showed a reduced content of Zea compared to WT, but no significant variation in other carotenoids was noted. The double mutant *szl1-1npq1-2* (impaired in lycopene β-cyclase and violaxanthin de-epoxidase) contained significantly lower contents of Ant, Vio and VAZ but significantly higher Lut content than WT Arabidopsis. In the *lut1* (impaired ε-ring hydroxylation) genotype the content of Lut was lower than in the WT.

Two-way ANOVA showed significant (*p* < 0.01) UV-induced changes in the content of carotenoids across genotypes and treatment (Figure 5 and Figure S7). Yet, many of the UV-induced changes are qualitatively similar to those observed in the WT. Thus, UV increases the carotenoid content in a similar manner in mutants as in WT. For instance, in the case of *aba1-6*, contents of Lut, Ant, β-Car and total carotenoids were significantly (*p* < 0.05) increased, resembling similar increases in the WT. Similarly, both *npq1-2* and *npq4-1* showed significant UV-mediated increases (*p* < 0.05) for Lut, Vio, Neo, VAZ and total carotenoid, resembling similar increases observed in the WT. In the case of *szl1-1npq1-2* no notable variation in carotenoid contents was found noted when comparing UV treated with untreated plants. Despite this, the contents of total carotenoids and Lut *in szl1-1npq1-2* were the highest in comparison to all other genotypes and the wild type.

**Figure 6:**
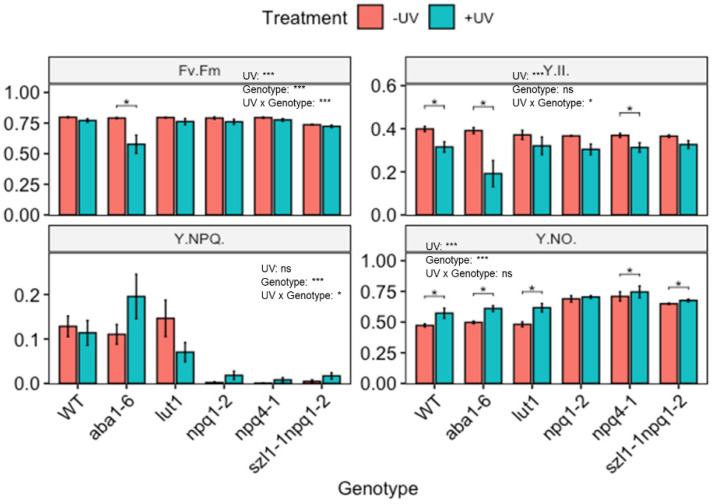
Photosynthetic efficiency of wild-type (WT) and carotenoid biosynthesis (*aba1-6*, *lut1*, *npq1-2*, *npq4-1* and *szl1-1npq1-2*) impaired *Arabidopsis thaliana* (Col-0). Plants were subjected to UV treatment for 3.75 h daily and harvested after 18 h on day 4. F_v_/F_m_: the maximum quantum efficiency of photosystem II (PSII); Y(II): the quantum efficiency of PSII under steady-state light conditions; Y(NO): yield of non-regulated non-photochemical energy dissipation and Y(NPQ): the yield of regulated non-photochemical energy dissipation. Bar plots show means and error bars indicate standard error for n = 5. The text in each panel shows the outcome of a two-way ANOVA across treatment and exposure duration. The stars above pairs of bars show the outcome of *t*-tests between the UV treatments. In both cases * (*p* < 0.05) and *** (*p* < 0.001) depict significant differences with ns depicting a non-significant difference.

To determine the consequences of changes in carotenoid contents, the photosynthetic efficiency of mutant plants was determined (Figure 6). Under control (non-UV) conditions, some carotenoid biosynthesis impaired genotypes showed significant decreases in photosynthetic efficiency compared to WT Arabidopsis. For example, the *szl1-1npq1-2* mutant displayed a significantly reduced F_v_/F_m_ compared to WT Arabidopsis. All other genotypes showed similar F_v_/F_m_ values as the untreated WT. The genotypes *npq1-2*, *npq4-1* and *szl1-1npq1-2* all showed dramatically reduced Y(NPQ) as well as a slight decrease in Y(II) and a significantly higher Y(NO) compared to the untreated WT Arabidopsis.

Two-way ANOVA showed that UV treatment caused significant changes in F_v_/F_m_, Y(II) and Y(NO) but not Y(NPQ). Genotype significantly affected F_v_/F_m_, Y(NPQ) and Y(NO). For instance, the WT Arabidopsis displayed a decrease in Y(II), and a slight increase in Y(NO) (Figure 6) following UV-exposure. Arabidopsis plants with impaired zeaxanthin de-epoxidase activity (*aba1-6*) showed a significantly reduced F_v_/F_m_ and Y(II) and increased Y(NO) in the UV exposed compared to untreated plants. An increase in Y(NPQ) was not statistically significant. In all other genotypes, effects of UV exposure were more modest. Genotypes lacking zeaxanthin de-epoxidase activity (*npq1-2*) and defective in pH-dependent NPQ (*npq4-1*) lack Y(NPQ) under non-UV conditions, however, a slight but non-significant quenching activity was noted in plants exposed to UV. Similarly, UV-treated *szl1-1npq1-2* displayed slightly higher Y(NO) than untreated controls.

## 4. Discussion

Abiotic environmental factors, such as temperature, drought, salinity, ozone, carbon dioxide and photosynthetic light, co-regulate plant carotenoid profiles [64]. Photosynthetically active radiation in particular controls the synthesis and accumulation of carotenoids, which in turn play a key role in, amongst others, photoprotection of the photosynthetic machinery [38,47,64]. However, relatively little is known about potential UV-induced alterations in the carotenoid profile, and any potential role in UV-B protection.

### 4.1. UV-Induced Accumulation of Xanthophyll Cycle Pigments

This study has shown that UV exposure induces changes in total leaf carotenoid content. The effect is most likely a UV-B effect, although it is noted that the UV treatment used in this study also contained a small additional amount of UV-A not present in the non-UV treatment (Table 2). Effects are most likely associated with the regulatory, rather than the stress-inducing, effects of UV radiation of plants, as no macroscopic damage was noted in UV treated plants, nor were substantial negative effects on photosynthesis measured. Results confirm the acclimatory UV-B response previously reported in the literature [14,65,66]. Specifically, this study noted accumulation of Ant, Neo, Vio, Lut, VAZ as well as total carotenoids in UV exposed Arabidopsis WT. A recent meta-analysis [23] highlighted that across the published literature the accumulation of Vio is the most common response to UV exposure. In contrast, a paper by Emiliani et al. [50] reported an increase in Zea, which was not observed in this study. A possible explanation for this dissimilar effect relates to the “double-edged” role of UV-B radiation, as regulator as well as stressor, depending on the applied dose [3]. In the current study, the UV dose was relatively low, and acclimatory responses seemed to be most prominent, including an increase in chlorophyll content, and a lack of inhibition of photosynthesis. Effects on F_v_/F_m_, indicating impairment of photosystem II were small, although a decrease in Y(II) was noted suggesting that UV affects photosynthesis at a site different from photosystem II. Largely “acclimatory” results were also obtained by Biswas et al. [65] who reported UV-induced changes in carotenoid profile in plants exposed to environmentally relevant doses of UV. In contrast, significant DNA damage and a reduction in photosynthetic efficiency were noted by Emiliani et al. [50] in plants exposed to UV. A role for stress in determining the balance between Vio and Zea induction remains unproven, yet a meta-analysis [23] has revealed that some of the variability in UV-induced changes in carotenoid profile can be linked to induction of stress.

An intriguing question is why UV-acclimation includes an increase in Vio content, a response typically associated with adjustment of the photosynthetic machinery to low light conditions. In this study, substantial increases in Vio were accompanied by modest increases in Ant, Neo and a non-significant increase in Zea, suggesting that the overall capacity of the xanthophyll cycle is upregulated in UV-exposed WT plants (as is evident from the increases in VAZ). If enough violaxanthin de-epoxidase activity is present to convert Vio into Zea [39], it can be hypothesised that UV exposure increases the capacity of the xanthophyll cycle, i.e., the capacity of plants to rapidly adjust to high visible light conditions. This hypothesis can be tested through UV supplementation studies using high background levels of PAR. If confirmed, the data imply that plants exploit UV radiation to adjust the photosynthetic machinery to high PAR intensity, an environmental condition that typically correlates with UV exposure.

### 4.2. UV-Induced Accumulation of Lutein

An increase in Lut accumulation is also noted in UV treated plants. Lutein is generally the carotenoid present at the highest content in plants, and contents tend to be relatively stable across a broad range of visible light conditions [39]. However, this does not appear to apply to UV radiation exposure. Biswas et al. [65] noted a positive correlation between Lut content and UV exposure conditions, including a correlation of Lut content with non-photochemical quenching in Arabidopsis. This increase in Lut in UV exposed plants likely relates to the ability of Lut to convert the energy of excited triplet chlorophyll to heat [67], thus contributing to photoprotection. The Lut epoxide cycle [36] has been hypothesised to involve the de-epoxidation of lutein epoxide into lutein as part of plant photoprotection [68], although this cycle is not thought to be present in Arabidopsis. The data presented in this study indicate that UV-acclimation involves upregulation of both the capacity of the xanthophyll cycle (VAZ) as well as the lutein content, supporting the hypothesis that plants exploit UV radiation as a signal to upregulate photoprotective responses that potentially help withstand exposure to high PAR intensity, that typically accompanies UV exposure.

### 4.3. The Role of Photoreceptors in the UV-Induced Accumulation of Carotenoids

UV-A and UV-B wavelengths are known to regulate the synthesis of some classes of plant metabolites via wavelength-specific photoreceptors (such as UVR8, phototropin, and cryptochrome) [69,70]. For example, biosynthesis of flavonoids and other phenolics is co-regulated through the photoreceptors UVR8 [12,16,71] and cryptochrome [72]. This study shows that the accumulation of carotenoids under UV radiation is not dependent on one particular photoreceptor. It appears that individual photoreceptors do not necessarily play a critical role in carotenoid induction. Previously, it was shown that accumulation of tocopherol does not depend on the functional photoreceptors UVR8, phototropin, or cryptochrome [20]. These data may be interpreted as meaning that UV drives carotenoid accumulation through other UV-induced processes, perhaps the inhibition of photosynthesis, or possibly through combinations of redundant photoreceptors [69]. Alternatively, the role of specific photoreceptors may be masked because of induction through other environmental factors. For example, Coffey et al. [16] showed a strong induction of UV-screening pigments in Arabidopsis cultured under low temperature, winter conditions, and this was noted in both WT and a *uvr8* deficient mutant. Thus, although the current study yields no evidence for a role of individual photoreceptors in the UV-mediated accumulation of carotenoids, such a role cannot be fully excluded. What is, however, clear is that both UVR8 and *phototropins* are important for UV protection of photosynthetic efficiency, but this is most likely an effect that does not involve carotenoids, but rather accumulation of UV screening pigments such as flavonoids.

### 4.4. Carotenoid Profile of Arabidopsis Plants with Impaired Carotenoid Biosynthesis

#### 4.4.1. Carotenoid Profiles under PAR

The accumulation of carotenoids in plants is reliant on a cascade of genes that encode the enzymes responsible for the synthesis of carotenes and xanthophylls. Many of these genes and gene-products have been studied [24] (Figure 1), and mutants with altered functionality of key genes are readily available. In most cases, these mutants display complex changes in carotenoid profile. For example, *npq1-2* and *npq4-1* Arabidopsis mutants are defective in violaxanthin de-epoxidase and pH-dependent non-photochemical quenching of chlorophyll fluorescence, respectively. Consistent with a lack of violaxanthin de-epoxidase activity, both *npq1-2* and *npq4-1* mutants showed reduced Zea contents in comparison to the WT Arabidopsis. As Zea is a key component in the non-photochemical quenching, the reduction in content is associated with particularly low values of Y(NPQ). These findings agree with what was reported in Niyogi et al. [54] where Zea contents in low PAR grown plants were reduced by as much as 60%. Similarly, *szl1-1npq1-2* is reported to be characterised by low Y(NPQ) and low contents of Vio and VAZ, but increased contents of Lut [27], presumably as a result of the re-direction of precursors in to the α-carotene branch of the carotenoid biosynthetic pathway. Again, the control data (PAR only) in this study are consistent with previous reports. Finally, the *Lut1* mutant characterised by impaired lutein synthesis showed somewhat reduced contents of Lut in comparison to the WT Arabidopsis as reported earlier [73]. The Lut genotype (*lut1)* contains low contents of Lut indicating a leaky-phenotype. Accompanying the decrease in Lut were changes in other carotenoids. For example, VAZ increased in *Lut1* and this can be explained as a compensatory characteristic, whereby restrictions in the metabolic pathway leading to Lut result in increased contents of Vio [37,73,74].

#### 4.4.2. The Carotenoid Profile of UV-Exposed Plants

In this report it is shown that UV induces increased accumulation of Ant, Neo, Vio, and Lut, but not β-Car and Zea in WT Arabidopsis. To explore the potential functionality of these changes in carotenoid profile, mutants with impaired biosynthetic pathways were used. It was found that the UV-induced accumulation of specific carotenoids was slightly altered in some mutants, compared to WT Arabidopsis. For example, there is a lack of UV-induced Vio accumulation in the *szl1-1npq1-2* line. Yet, despite a low Vio content, the F_v_/F_m_ and Y(II) values for UV exposed *szl1-1npq1-2* are similar to those of Arabidopsis WT, indicating that Vio and the xanthophyll cycle do not play a key role in protecting the photosynthetic machinery from UV radiation.

**Figure 7:**
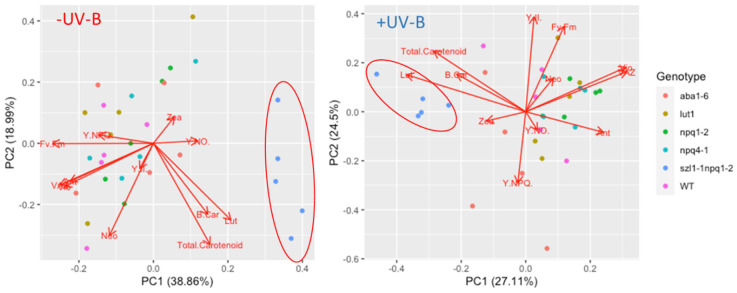
Principal component analysis showing the distribution of UV treated and/or control carotenoid impaired Arabidopsis genotypes (score plot), based on the influence of carotenoids and photosynthetic efficiency parameters (loading plot).

A more detailed PCA-based analysis of the relationships between individual carotenoids and photosynthetic parameters shows that in control plants there is an overall association between Y(NPQ), VAZ, Vio and Ant, which also aligns with overall photosynthetic efficiency of photosystem II (F_v_/F_m_ and Y(II)) (Figure 7). The link between xanthophyll pigments and Y(NPQ) is well established in the scientific literature [32,47]. Interestingly, exposure to UV-B and short wavelength UV-A disrupts the association between Y(NPQ) and VAZ, Vio, and Ant in UV exposed plants (Figure 7). The UV-induced increase in Vio reported in this study does lead to an overall increase in VAZ but is not associated with non-photochemical quenching. One explanation for the decoupling of Vio and VAZ from non-photochemical quenching relates to the reported inhibition of violaxanthin de-epoxidase activity in UV-B exposed plants [49], impeding the formation of Zea. Yet, it is intriguing that Vio and VAZ are loosely associated with both F_v_/F_m_ and Y(II) in UV-exposed plants, while Y(NPQ) is not. It could be speculated that these data imply a novel role for Vio in UV protection that does not relate directly to the xanthophyll cycle and non-photochemical quenching. However, it is more likely that the trend in Vio and VAZ is statistically correlated with other (not measured) acclimatory UV responses such as an increase in total antioxidant activity and/or content of UV screening pigments which are well documented to contribute to protection of the photosynthetic machinery from UV radiation [4]. Yet, a lack of a direct role for Vio in UV protection leaves open the question why so many studies [23] show the UV-induced accumulation of this xanthophyll pigment. Recent studies indicate that plants exploit UV radiation as a proxy for other environmental stressors such as drought [75]. Neo in particular is closely linked to ABA synthesis which, in turn, affects many aspects of plant physiology including stomatal closure, as well as cross-resistance to a wide variety of biotic and abiotic stressors. Indeed, a study of grapevine leaves showed that cumulative UV radiation exposure over a large geographical gradient was the strongest correlator with accumulation of a broad range of metabolites and pigments [76]. Thus, the possibility that the primary function of UV-induced alterations in the carotenoid profile is not UV protection, but rather protection against other environmental stressors, should be considered. Consequently, future studies on the functional role of UV-induced carotenoids need to be performed under conditions of high PAR.

## 5. Conclusions

Overall, this study has shown that UV radiation induces specific changes in the carotenoid profile of *Arabidopsis thaliana*. Specifically, content of antheraxanthin, neoxanthin, violaxanthin, and lutein increased in UV-exposed leaves. This acclimatory UV response was found not to require a functional UV-B (UVR8) or UV-A (Cryptochrome and Phototropin) photoreceptor. Remarkably, UV-induced accumulation of carotenoids such as violaxanthin could not be linked to protection of the photosynthetic machinery from UV damage, triggering a question about the functional relevance of this UV response. Here, it is argued that plants exploit UV radiation as a proxy for other stressors. Thus, it is speculated that the primary function of UV-induced alterations in the carotenoid profile is not UV protection, but rather protection against other environmental stressors such as accompanying high PAR intensity.

## Figures and Tables

**Table 1:** List and details of carotenoid impaired genotypes used in this study. Key enzymes are as indicated in Figure 1.

Mutants and (NASC Code)	Mutant Name	Target Gene/Enzyme	Deficiency/Function	References Detailing Mutants
*aba1 (npq2)* *N3772*	*ABA-DEFICIENT-1*	Zeaxanthin epoxidase	ABA deficiency	[54]
*lut1/salk_097275c* N655128	*LUTEIN-1*	Lycopene ε cyclase	Lutein deficiency	[55]
*npq1-2* N69600	*NON-PHOTOCHEMICAL QUENCHING 1*	Violaxanthin de-epoxidase	Reduced nonphotochemical quenching due to the absence of zeaxanthin formation	[54]
*npq4* N66021	*NON-PHOTOCHEMICAL QUENCHING 4*	Chlorophyll a-b binding family protein	Defective in pH-dependent npq of chlorophyll fluorescence	[56]
*szl1-1npq1-2* N66023	*SUPRESOR OF ZEAXANTHIN-LESS 1-NON-PHOTOCHEMICAL QUENCHING 1*	Lycopene β-cyclase/violaxanthin de-epoxidase	Restores npq defects of npq1-2 mutant.	[57]

**Table 2:** Light conditions used in experiments. The biological effective daily dose was calculated using the action spectra formulae Gen-T [59].

Treatment	Filter Used	PAR	UV-B	UV-A	Biologically Effective Dose	
					UV-B	UV-A
−UV	Mylar	188 µmol m^−2^ s^−1^	0.00 W m^−2^	0.25 W m^−2^	0.14 kJ m^−2^	0.18 kJ m^−2^
+UV	Cellulose acetate	188 µmol m^−2^ s^−1^	0.22 W m^−2^	0.37 W m^−2^	2.53 kJ m^−2^	0.28 kJ m^−2^

## Data Availability

The data that support the findings of this study are available in GitHub at https://github.com/LanreBadmus/UV_Carotenoid_Induction (accessed on 13 December 2022).

## Supplementary Materials

The following supporting information can be downloaded at: https://www.mdpi.com/article/10.3390/biom12121879/s1, Figure S1; Chlorophyll levels (µg·g^−1^ FW) of wild-type *Arabidopsis thaliana* (Col-0) exposed to varying durations of UV; Figure S2: Carotenoid contents of wild type (WT) and photoreceptor (cryptochrome (*cry1cry2*), phototropin (*phot1phot2*) and *uvr8*) deficient *Arabidopsis thaliana* (Col-0); Figure S3: Chlorophyll levels (µg·g^−1^ FW) of wild type (WT) and photoreceptor (cryptochrome (*cry1cry2*), phototropin (*phot1phot2*) and *uvr8*) deficient *Arabidopsis thaliana* (Col-0). Figure S4: Chlorophyll levels (µg·g^−1^ FW) of wild type (WT) and carotenoid biosynthesis (*aba1-6*, *lut1*, *lut2*, *npq1-2*, *npq4-1*, *szl1-1* and *szl1-1npq1-2*) impaired *Arabidopsis thaliana* (Col-0).

## References

[1] Smith H. (1982). Light Quality, Photoperception, and Plant Strategy. Annu. Rev. Plant Physiol. Plant Mol. Biol..

[2] Huché-Thélier L., Crespel L., Le Gourrierec J., Morel P., Sakr S., Leduc N. (2016). Light signaling and plant responses to blue and UV radiations-Perspectives for applications in horticulture. Environ. Exp. Bot..

[3] Hideg É., Jansen M.A.K., Strid Å. (2013). UV-B exposure, ROS, and stress: Inseparable companions or loosely linked associates?. Trends Plant Sci..

[4] Jansen M.A.K., Gaba V., Greenberg B.M. (1998). Higher plants and UV-B radiation: Balancing damage, repair and acclimation. Trends Plant Sci..

[5] Schreiner M., Martínez-Abaigar J., Glaab J., Jansen M.A.K. (2014). UV-B Induced Secondary Plant Metabolites. Opt. Photonik.

[6] Jenkins G.I. (2017). Photomorphogenic responses to ultraviolet-B light. Plant Cell Environ..

[7] Tossi V.E., Regalado J.J., Iannicelli J., Laino L.E., Burrieza H.P., Escandón A.S., Pitta-Álvarez S.I. (2019). Beyond Arabidopsis: Differential UV-B response mediated by UVR8 in diverse species. Front. Plant Sci..

[8] Klem K., Holub P., Štroch M., Nezval J., Špunda V., Tříska J., Jansen M.A.K., Robson T.M., Urban O. (2015). Ultraviolet and photosynthetically active radiation can both induce photoprotective capacity allowing barley to overcome high radiation stress. Plant Physiol. Biochem..

[9] Hideg É., Strid A., Jordan B. (2017). The effects of UV-B on the biochemistry and metabolism of plants. UV-B Radiation and Plant Life: Molecular Biology to Ecology.

[10] Vandenbussche F., Yu N., Li W., Vanhaelewyn L., Hamshou M., Van Der Straeten D., Smagghe G. (2018). An ultraviolet B condition that affects growth and defense in Arabidopsis. Plant Sci..

[11] Verdaguer D., Jansen M.A.K., Llorens L., Morales L.O., Neugart S. (2017). Plant Science UV-A radiation effects on higher plants: Exploring the known unknown. Plant Sci..

[12] Brown B.A., Cloix C., Jiang G.H., Kaiserli E., Herzyk P., Kliebenstein D.J., Jenkins G.I. (2005). A UV-B-specific signaling component orchestrates plant UV protection. Proc. Natl. Acad. Sci. USA.

[13] Morales L.O., Tegelberg R., Brosché M., Keinänen M., Lindfors A., Aphalo P.J. (2010). Effects of solar UV-A and UV-B radiation on gene expression and phenolic accumulation in Betula pendula leaves. Tree Physiol..

[14] Hectors K., Van Oevelen S., Geuns J., Guisez Y., Jansen M.A.K., Prinsen E. (2014). Dynamic changes in plant secondary metabolites during UV acclimation in Arabidopsis thaliana. Physiol. Plant..

[15] Neugart S., Fiol M., Schreiner M., Rohn S., Zrenner R., Kroh L.W., Krumbein A. (2014). Interaction of moderate UV-B exposure and temperature on the formation of structurally different flavonol glycosides and hydroxycinnamic acid derivatives in kale (Brassica oleracea var. sabellica). J. Agric. Food Chem..

[16] Coffey A., Prinsen E., Jansen M.A.K., Conway J. (2017). The UVB photoreceptor UVR8 mediates accumulation of UV-absorbing pigments, but not changes in plant morphology, under outdoor conditions. Plant Cell Environ..

[17] Celeste Dias M., Pinto D.C.G.A., Correia C., Moutinho-Pereira J., Oliveira H., Freitas H., Silva A.M.S., Santos C. (2018). UV-B radiation modulates physiology and lipophilic metabolite profile in Olea europaea. J. Plant Physiol..

[18] Contreras R.A., Pizarro M., Köhler H., Zamora P., Zúñiga G.E. (2019). UV-B shock induces photoprotective flavonoids but not antioxidant activity in Antarctic Colobanthus quitensis (Kunth) Bartl. Environ. Exp. Bot..

[19] Liu Y., Liu J., Wang H.-Z., Wu K.-X., Guo X.-R., Mu L.-Q., Tang Z.-H. (2020). Comparison of the global metabolic responses to UV-B radiation between two medicinal Astragalus species: An integrated metabolomics strategy. Environ. Exp. Bot..

[20] Badmus U.O., Crestani G., Connell R.D.O., Cunningham N., Jansen M.A.K. (2022). UV-B induced accumulation of tocopherol in Arabidopsis thaliana is not dependent on individual UV photoreceptors. Plant Stress.

[21] Vogt T. (2010). Phenylpropanoid biosynthesis. Mol. Plant.

[22] Jansen M.A.K., Bilger W., Hideg E., Strid Å., Participants U.W., Urban O. (2019). Interactive effects of UV-B radiation in a complex environment. Plant Physiol. Biochem..

[23] Badmus U.O., Alexander A., Klem K., Urban O., Jansen M.A.K. (2022). A meta-analysis of the effects of UV radiation on the plant carotenoid pool. Plant Physiol. Biochem..

[24] Britton G. (1995). Structure and properties of carotenoids in relation to function. FASEB J..

[25] Fraser P.D., Bramley P.M. (2004). The biosynthesis and nutritional uses of carotenoids. Prog. Lipid Res..

[26] Britton G., Liaaen-Jensen S., Pfander H., Britton G., Liaaen-Jensen S., Pfander H. (2009). Carotenoids Special Molecules, Special Properties. Nutrition and Health.

[27] Cazzaniga S., Bressan M., Carbonera D., Agostini A., Dall’osto L. (2016). Differential Roles of Carotenes and Xanthophylls in Photosystem I Photoprotection. Biochemistry.

[28] Niyogi K.K., Björkman O., Grossman A.R. (1997). The roles of specific xanthophylls in photoprotection. Proc. Natl. Acad. Sci. USA.

[29] Havaux M. (1998). Carotenoids as membrane stabilizers in chloroplasts. Trends Plant Sci..

[30] Xiao F.G., Shen L., Ji H.F. (2011). On photoprotective mechanisms of carotenoids in light harvesting complex. Biochem. Biophys. Res. Commun..

[31] Pogson B.J., Rissler H.M. (2000). Genetic manipulation of carotenoid biosynthesis and photoprotection. Philos. Trans. R. Soc. B Biol. Sci..

[32] Demmig-Adams B., Cohu C.M., Stewart J.J., Iii W.W.A., Demmig-Adams B., Garab G., Adams W., III (2014). Non-Photochemical Quenching and Energy Dissipation in Plants, Algae and Cyanobacteria. Photosynthesis and Respiration.

[33] Bykowski M., Mazur R., Wo J., Suski S. (2021). Too rigid to fold: Carotenoid-dependent decrease in thylakoid fluidity hampers the formation of chloroplast grana. Plant Physiol..

[34] Son M., Pinnola A., Gordon S.C., Bassi R., Schlau-Cohen G.S. (2020). Observation of dissipative chlorophyll-to-carotenoid energy transfer in light-harvesting complex II in membrane nanodiscs. Nat. Commun..

[35] Umena Y., Kawakami K., Shen J., Kamiya N. (2011). Crystal structure of oxygen-evolving photosystem II at a resolution of 1.9 Å. Nature.

[36] Jahns P., Holzwarth A.R. (2012). The role of the xanthophyll cycle and of lutein in photoprotection of photosystem II. Biochim. Biophys. Acta—Bioenerg..

[37] Xu P., Chukhutsina V.U., Nawrocki W.J., Schansker G., Bielczynski L.W., Lu Y., Karcher D., Bock R., Croce R. (2020). Photosynthesis without b -carotene. Elife.

[38] Demmig-Adams B. (1990). Carotenoids and photoprotection in plants: A role for the xanthophyll zeaxanthin. Biochim. Biophys. Acta—Bioenerg..

[39] Demmig-Adams B., Stewart J.J., López-Pozo M., Polutchko S.K., Adams W.W. (2020). Zeaxanthin, a Molecule for Photoprotection in Many Different Environments. Molecules.

[40] Ablazov A., Mi J., Jamil M., Jia K.P., Wang J.Y., Feng Q., Al-Babili S. (2020). The Apocarotenoid Zaxinone Is a Positive Regulator of Strigolactone and Abscisic Acid Biosynthesis in Arabidopsis Roots. Front. Plant Sci..

[41] Müller M., Munné-Bosch S. (2021). Hormonal impact on photosynthesis and photoprotection in plants. Plant Physiol..

[42] Pang X., Zhang Z., Wen X., Ban Y., Moriguchi T., Yusuke Y. (2007). Polyamines, all-purpose players in response to environment stresses in plants. Plant Stress.

[43] Moreno J.C., Mi J., Alagoz Y., Al-Babili S. (2021). Plant apocarotenoids: From retrograde signaling to interspecific communication. Plant J..

[44] Llorente B., Martinez-Garcia J.F., Stange C., Rodriguez-Concepcion M. (2017). Illuminating colors: Regulation of carotenoid biosynthesis and accumulation by light. Curr. Opin. Plant Biol..

[45] Heath J.J., Cipollini D.F., Stireman J.O. (2013). The role of carotenoids and their derivatives in mediating interactions between insects and their environment. Arthropod-Plant Interact..

[46] García-Chavarría M., Lara-Flores M. (2013). The use of carotenoid in aquaculture. Res. J. Fish. Hydrobiol..

[47] Havaux M., Niyogi K.K. (1999). The violaxanthin cycle protects plants from photooxidative damage by more than one mechanism. Proc. Natl. Acad. Sci. USA.

[48] Jansen M.A.K., Hectors K., O’Brien N.M., Guisez Y., Potters G. (2008). Plant stress and human health: Do human consumers benefit from UV-B acclimated crops?. Plant Sci..

[49] Pfündel E.E., Pan R.S., Dilley R.A. (1992). Inhibition of violaxanthin deepoxidation by ultraviolet-B radiation in isolated chloroplasts and intact leaves. Plant Physiol..

[50] Emiliani J., D’Andrea L., Falcone Ferreyra M.L., Maulión E., Rodriguez E., Rodriguez-Concepción M., Casati P. (2018). A role for β,β-xanthophylls in Arabidopsis UV-B photoprotection. J. Exp. Bot..

[51] Favory J.J., Stec A., Gruber H., Rizzini L., Oravecz A., Funk M., Albert A., Cloix C., Jenkins G.I., Oakeley E.J. (2009). Interaction of COP1 and UVR8 regulates UV-B-induced photomorphogenesis and stress acclimation in Arabidopsis. EMBO J..

[52] Mao J., Zhang Y.C., Sang Y., Li Q.H., Yang H.Q. (2005). A role for Arabidopsis cryptochromes and COP1 in the regulation of stomatal opening. Proc. Natl. Acad. Sci. USA.

[53] Kinoshita T., Doi M., Suetsugu N., Kagawa T., Wada M., Shimazaki K. (2001). phot1 and phot2 mediate blue light regulation of stomatal opening. Nature.

[54] Niyogi K.K., Grossman A.R., Björkman O. (1998). Arabidopsis mutants define a central role for the xanthophyll cycle in the regulation of photosynthetic energy conversion. Plant Cell.

[55] Alonso J.M., Stepanova A.N., Leisse T.J., Kim C.J., Chen H., Shinn P., Stevenson D.K., Zimmerman J., Barajas P., Cheuk R. (2003). Genome-wide insertional mutagenesis of Arabidopsis thaliana. Science.

[56] Li X.P., Björkman O., Shih C., Grossman A.R., Rosenquist M., Jansson S., Niyogi K.K. (2000). A pigment-binding protein essential for regulation of photosynthetic light harvesting. Nature.

[57] Li Z., Ahn T.K., Avenson T.J., Ballottari M., Cruz J.A., Kramer D.M., Bassi R., Fleming G.R., Keasling J.D., Niyogi K.K. (2009). Lutein accumulation in the absence of zeaxanthin restores nonphotochemical quenching in the arabidopsis thaliana npq1 mutant. Plant Cell.

[58] Boyes D.C., Zayed A.M., Ascenzi R., McCaskill A.J., Hoffman N.E., Davis K.R., Görlach J. (2001). Growth stage-based phenotypic analysis of Arabidopsis: A model for high throughput functional genomics in plants. Plant Cell.

[59] Aphalo P.J., Albert A., Björn L.O., Mcleod A.R., Robson T.M., Rosenqvist E. (2012). Beyond the Visible: A handbook of best practice in plant UV photobiology. COST Action FA0906 UV4growth (Issue June 2014).

[60] Klughammer C., Schreiber U. (2008). Complementary PS II quantum yields calculated from simple fluorescence parameters measured by PAM fluorometry and the Saturation Pulse method. PAM Appl. Notes.

[61] Thayer S.S., Björkman O. (1990). Leaf Xanthophyll content and composition in sun and shade determined by HPLC. Photosynth. Res..

[62] Fraser P.D., Pinto M.E., Holloway D.E., Bramley P.M. (2000). Application of high-performance liquid chromatography with photodiode array detection to the metabolic profiling of plant isoprenoids. Plant J..

[63] Venables W.N., Ripley B.D. (2013). Modern Applied Statistics with S-Plus.

[64] Esteban R., Barrutia O., Artetxe U., Fernández-Marín B., Hernández A., García-Plazaola J.I. (2015). Internal and external factors affecting photosynthetic pigment composition in plants: A meta-analytical approach. New Phytol..

[65] Biswas D.K., Ma B.L., Xu H., Li Y., Jiang G. (2020). Lutein-mediated photoprotection of photosynthetic machinery in Arabidopsis thaliana exposed to chronic low ultraviolet-B radiation. J. Plant Physiol..

[66] Lee J.-H., Shibata S., Goto E. (2021). Time-Course of Changes in Photosynthesis and Secondary Metabolites in Canola (Brassica napus) Under Different UV-B Irradiation Levels in a Plant Factory With Artificial Light. Front. Plant Sci..

[67] Moreira-Rodríguez M., Nair V., Benavides J., Cisneros-Zevallos L., Jacobo-Velázquez D.A. (2017). UVA, UVB light, and methyl jasmonate, alone or combined, redirect the biosynthesis of glucosinolates, phenolics, carotenoids, and chlorophylls in broccoli sprouts. Int. J. Mol. Sci..

[68] García-Plazaola J.I., Hernández A., Olano J.M., Becerril J.M. (2003). The operation of the lutein epoxide cycle correlates with energy dissipation. Funct. Plant Biol..

[69] Rai N., Morales L.O., Aphalo P.J. (2021). Perception of solar UV radiation by plants: Photoreceptors and mechanisms. Plant Physiol..

[70] Ulm R., Jenkins G.I. (2015). Q&A: How do plants sense and respond to UV-B radiation?. BMC Biol..

[71] Clayton W.A., Albert N.W., Thrimawithana A.H., McGhie T.K., Deroles S.C., Schwinn K.E., Warren B.A., McLachlan A.R.G., Bowman J.L., Jordan B.R. (2018). UVR8-mediated induction of flavonoid biosynthesis for UVB tolerance is conserved between the liverwort Marchantia polymorpha and flowering plants. Plant J..

[72] Brelsford C.C., Morales L.O., Nezval J., Kotilainen T.K., Hartikainen S.M., Aphalo P.J., Robson T.M. (2019). Do UV-A radiation and blue light during growth prime leaves to cope with acute high light in photoreceptor mutants of Arabidopsis thaliana?. Physiol. Plant..

[73] Pogson B., McDonald K.A., Truong M., Britton G., DellaPenna D. (1996). Arabidopsis carotenoid mutants demonstrate that lutein is not essential for photosynthesis in higher plants. Plant Cell.

[74] Cazzaniga S., Li Z., Niyogi K.K., Bassi R., Dall’Osto L. (2012). The Arabidopsis szl1 mutant reveals a Critical role of β-carotene in photosystem I photoprotection. Plant Physiol..

[75] Jansen M.A.K., Ač A., Klem K., Urban O. (2022). A meta-analysis of the interactive effects of UV and drought on plants. Plant Cell Environ..

[76] Castagna A., Csepregi K., Neugart S., Zipoli G., Večeřová K., Jakab G., Jug T., Llorens L., Martínez-Abaigar J., Martínez-Lüscher J. (2017). Environmental plasticity of Pinot noir grapevine leaves: A trans-European study of morphological and biochemical changes along a 1,500-km latitudinal climatic gradient. Plant Cell Environ..

