# Supplementary material for: UV Radiation Induces Specific Changes in the Carotenoid Profile of Arabidopsis thaliana
Source: Biomolecules. 2022 Dec 14;12(12):1879. doi: 10.3390/biom12121879 (PMC9775031; doi:10.3390/biom12121879)
Supplement: Supplementary file 1 [file biomolecules-12-01879-s001.zip › biomolecules-2082278-supplementary.pdf]

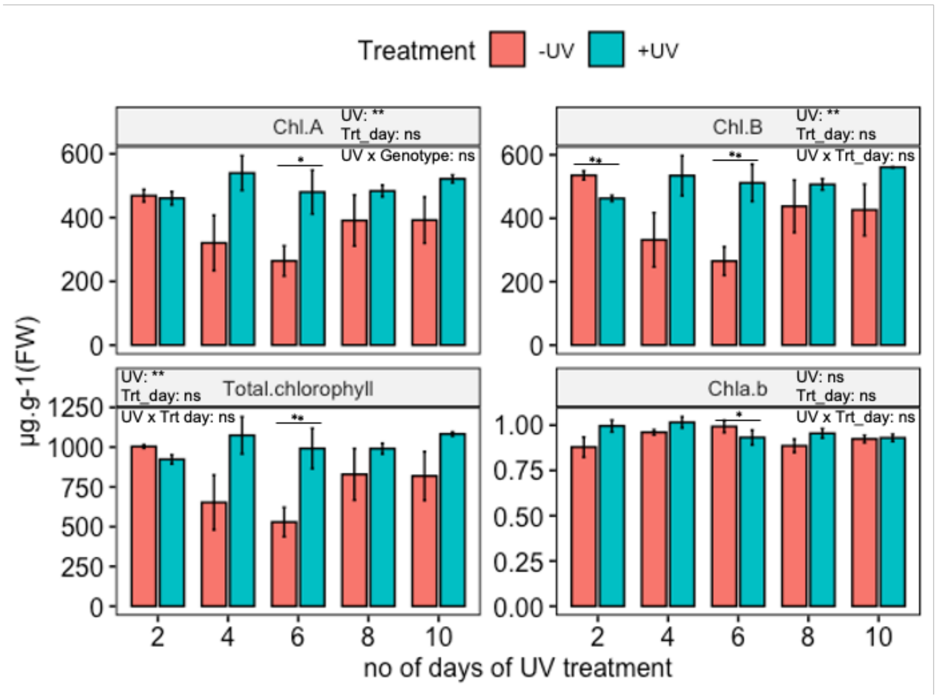

Figure S1: Chlorophyll levels (µg. g^{-1} FW) of wild-type *Arabidopsis thaliana* (Col-0) exposed to varying durations of UV. Plants were subjected to UV treatment for 3.75 hrs daily and harvested after 18 hours on days 2, 4, 6, 8 and 10. Bar plots show means and error bars indicate standard error for n = 5. 0.05* and 0.01** depict significant differences and ns depict non-significant differences, between treatments.

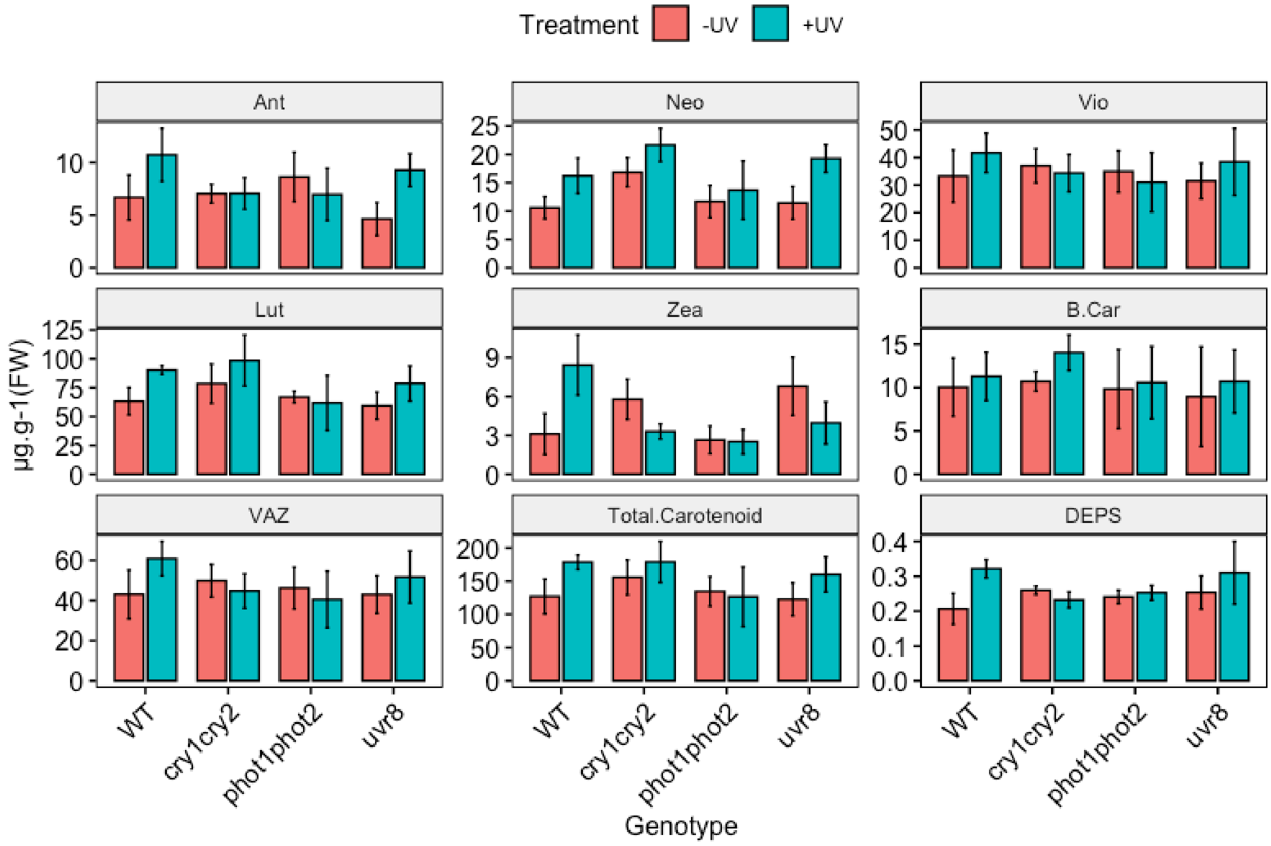

Figure S2: Carotenoid levels (µg. g^{-1} FW) of wild type (WT) and photoreceptor (cryptochrome (cry1cry2), phototropin (phot1phot2) and UVR8) deficient *Arabidopsis thaliana* (Col-0). Plants were subjected to UV treatment for 3.75 hrs daily and harvested after 18 hours on day 4. Bar plots show means and error bars indicate standard error for n = 5.

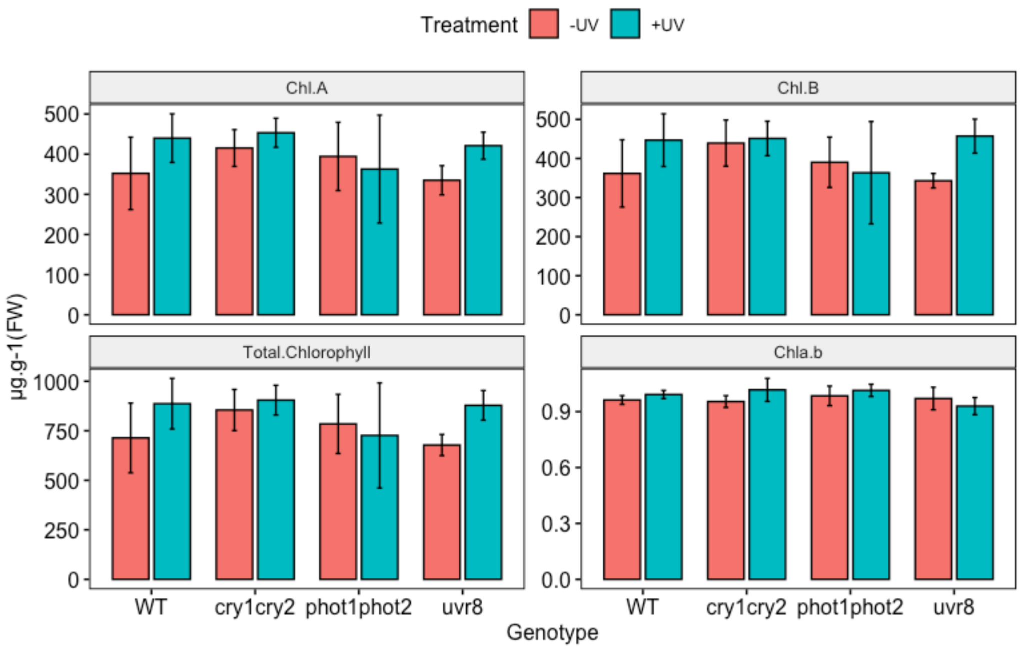

Figure S3: Chlorophyll levels (µg. g^{-1} FW) of wild type (WT) and photoreceptor (cryptochrome (cry1cry2), phototropin (phot1phot2) and UVR8) deficient *Arabidopsis thaliana* (Col-0). Plants were subjected to UV treatment for 3.75 hrs daily and harvested after 18 hours on day 4. Bar plots show means and error bars indicate standard error for n = 5.

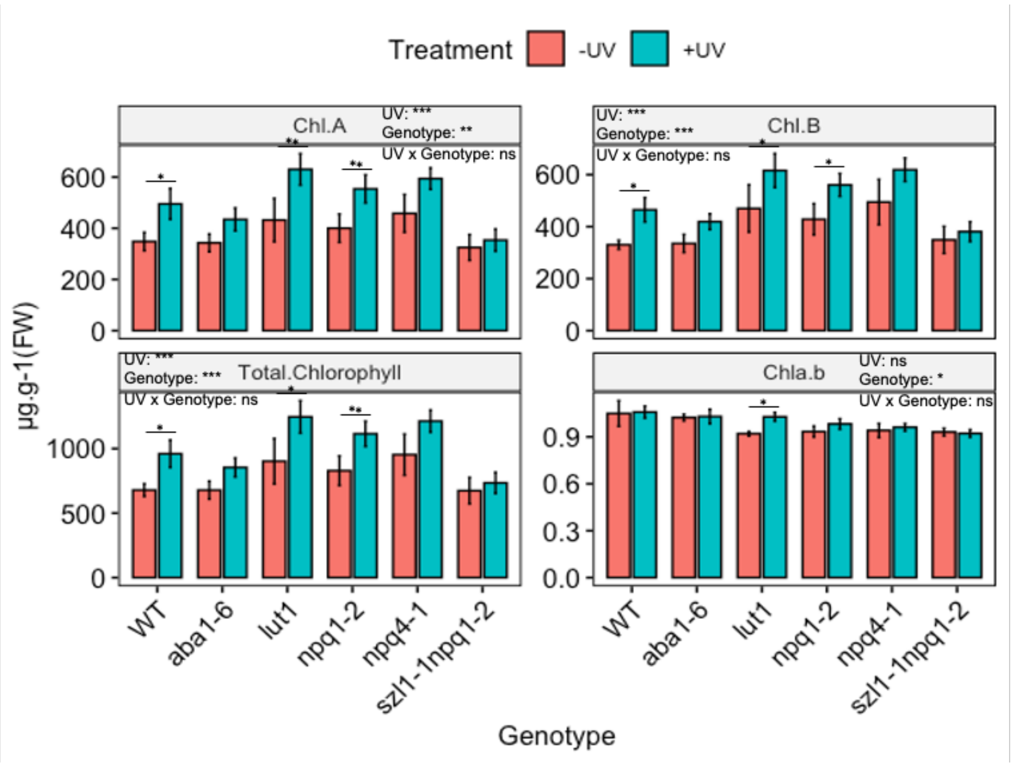

Figure S4: Chlorophyll levels (µg. g^{-1} FW) of wild type (WT) and carotenoid biosynthesis (*aba1-6, lut1, lut2, npq1-2, npq4-1, szl1-1* and *szl1-1npq1-2*) impaired *Arabidopsis thaliana* (Col-0) mutants. Plants were subjected to UV treatment for 3.75 hrs daily and harvested after 18 hours on day 4. Bar plots show means and error bars indicate standard error for n = 5. 0.05*, 0.01** and 0.001*** depict significant differences and ns depicts non-significant difference, between treatments.
